# Synthetic microbial communities for sustainable hydroponic tomato production

**DOI:** 10.1038/s44264-026-00147-8

**Published:** 2026-05-26

**Authors:** Samuel W. Wilkinson, Harry C. Wright, T. E. Anne Cotton, David Pascual-Pardo, Stuart A. Campbell, Upuli Wickramaarachchi, Duncan D. Cameron, Boglarka Z. Gulyas, Peter Ho, Alan R. Mackie, Samantha J. Caton, Jurriaan Ton, Stephen A. Rolfe

**Affiliations:** 1 Plants, Photosynthesis and Soil, School of Biosciences, The University of Sheffield, Sheffield, United Kingdom; 2 Biology Department, Centre for Novel Agricultural Products (CNAP), University of York, York, United Kingdom; 3 School of Mathematical and Physical Sciences, The University of Sheffield, Sheffield, United Kingdom; 4 Manchester Institute of Biotechnology, University of Manchester, Manchester, United Kingdom; 5 Department of Earth and Environmental Sciences, School of Natural Sciences, University of Manchester, Manchester, United Kingdom; 6 Sheffield Centre for Health and Related Research, Division of Population Health, School of Medicine and Population Health, The University of Sheffield, Sheffield, United Kingdom; 7 School of Food Science and Nutrition, University of Leeds, Leeds, United Kingdom

**Keywords:** Biotechnology, Microbiology, Plant sciences

## Abstract

Hydroponic horticulture will play a key role in future food production as the growing global population becomes increasingly urbanised. Tomato (*Solanum lycopersicum*) is a widely grown and consumed crop that is already cultivated hydroponically in glasshouses in areas of the world with cooler climates, such as Northern Europe. Hydroponic growing systems enable high yields but can enhance disease susceptibility which increases the risk of devastating yield losses. Manipulation of the hydroponic microbiome has been proposed as a strategy to protect plants against disease. However, this hypothesis remains largely untested. We examined whether introducing synthetic communities of plant-beneficial microbes (SynComs) could offer a sustainable disease protection solution for hydroponic tomato production. We identified individual microbes and in turn two SynComs that induce systemic disease resistance during the vulnerable early stages of development. The two SynComs were evaluated further in a commercial-scale greenhouse trial. Although both SynComs reduced early growth, they had no adverse effects on yield or fruit quality. Strikingly, while only one SynCom strain consistently persisted in the hydroponic stone wool substrate throughout the six-month trial, the introduction of disease-suppressive SynComs at sowing had significant and similar impacts on bacterial community structure six months later. Our findings demonstrate that microbial SynComs can reduce disease susceptibility of hydroponically grown tomato without compromising yield, offering a viable and sustainable approach for crop protection in controlled environment agriculture.

## Introduction

Hydroponic cultivation involves growing plants using soilless support systems and a nutrient-rich water supply. These cropping systems drive an economically important global industry, which is predicted to more than double in value by 2030 to US$ 12.8 Billion^1^. This expansion is based on the growth and increased urbanisation of the human population, which is reducing the availability of agricultural land and increasing food demand and insecurity^2,3^. The ability to grow plants at high densities in urban environments, such as warehouses, brings food production closer to the consumer, while also reducing problems such as water availability, long-distance transport, soil erosion, and climate variability^4^. Tomato (*Solanum lycopersicum*) is one of the crops that is most widely cultivated in hydroponic growing systems, accounting for the largest revenue share of any crop in 2023^5^.

Although hydroponic tomato cultivation is highly productive^3^, limitations include high energy requirements and vulnerability of hydroponically grown tomato to disease^6^. Hydroponically grown crops are particularly vulnerable to waterborne diseases, which can easily spread through the highly interconnected water-based systems^7,8^. In the case of tomato, examples of problematic root-infecting diseases include crown and root rot (*Fusarium oxysporum*), bacterial wilt (*Ralstonia solanacearum*), damping off (*Globisporangium* sp.) and hairy root (*Agrobacterium* biovar 1 strains)^9–11^. Additionally, foliar disease, such as powdery mildew (*Oidium neolycopersici*) and bacterial speck (*Pseudomonas syringae*) cause major yield losses^12^. Traditional control measures rely on excessive sanitation, cultivation practices and pesticides^8,9,11^. However, many pesticides are being phased out due to increased regulatory restrictions on their use^13^. Biocontrol microbes have therefore been proposed as a sustainable alternative to control plant diseases in controlled environment horticulture^14^.

In soil, beneficial rhizosphere-inhabiting microorganisms suppress disease in plants via a variety of mechanisms^15^. Rhizosphere microbes can directly suppress disease via the production of compounds that are biocidal. For example, *Pseudomonas chlororaphis* PCL1391, isolated from the rhizosphere of tomato, produces the anti-fungal compound phenazine-1-carboxamide, which enables it to suppress foot and root rot disease in tomato caused by the fungal pathogen *Fusarium oxysporum* f. sp. *radicis-lycopersici*^16^. Rhizosphere microbes can also suppress diseases via indirect mechanisms, such as competition for space and nutrients or induced systemic resistance (ISR) in host plants^15^. ISR is the only mechanism that provides protection against both foliar and root pathogens^17^. Thus, introducing ISR-eliciting biocontrol microbes into hydroponic systems could provide long-lasting, broad-spectrum protection of crops against both below- and aboveground diseases.

Microbial-based biopesticides are already available for hydroponic horticulture and agriculture more widely, with many more in development^14,18,19^. However, single-strain inoculants, which have been developed and tested in laboratory environments, often have underwhelming benefits in commercial cultivation systems^8,20^. This has been linked to a multitude of factors including variation in genotype between crop varieties, substrate conditions (e.g. pH) and (a)biotic stresses. To overcome these issues, it has been proposed that synthetic communities (SynComs) consisting of multiple plant-beneficial microbes may be more reliable and consistent in disease-suppressive activity^21,22^. However, while the use of plant-beneficial SynComs has begun to be explored in soil systems, their benefits and long-term impacts on hydroponically grown crops remain unknown^8^.

In this study, we assessed the potential for SynComs as a viable tool for sustainable disease management in commercial horticulture. We screened beneficial microbes, applied at sowing, for their ability to protect hydroponic tomatoes against bacterial speck disease via ISR and to promote plant growth. The most promising strains were assembled into small SynComs to enhance reliability and performance. These SynComs were then evaluated under commercial-scale greenhouse conditions for their effects on key agronomic traits of tomato, post-harvest disease resistance and their long-term influence on the hydroponic microbiome.

## Results

### Hydroponically grown tomato is highly susceptible to disease

**Fig. 1:**
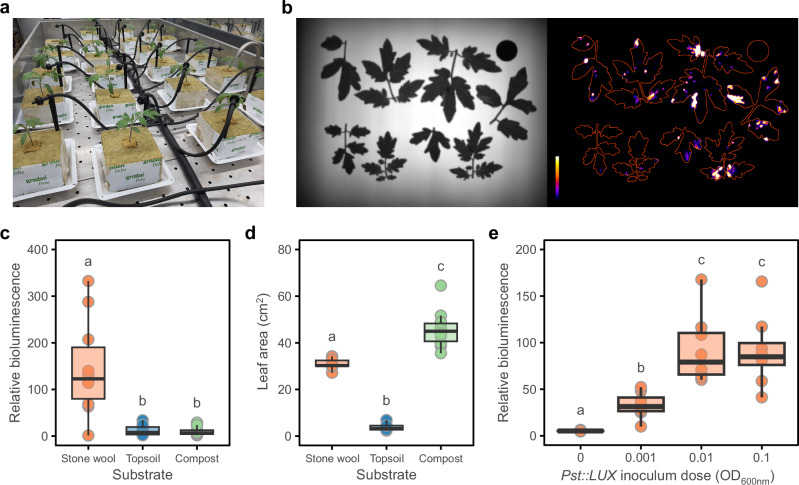
Effect of growing tomato in stone wool on disease susceptibility. **a** Tomato plants (cv. Moneymaker) growing hydroponically under laboratory-based controlled environment conditions. **b** Example images from assay for quantifying colonisation of tomato leaves by bioluminescent *Pseudomonas syringae* pv. *tomato* D3000 expressing the *luxCDABE* operon from *Photorhabdus luminescens* (*Pst::LUX*). Left-hand image, captured with illumination from below, provides leaf outlines for right-hand image taken in complete darkness with the only light source being bioluminescence from *Pst::LUX*. White and black equate to high and low relative bioluminescence, respectively. Red lines depict leaf outlines. **c**, **d** *Pst::LUX* colonisation, quantified at 3 days post inoculation, and leaf area of 19-day-old tomato plants grown in three different substrates (*n* = 9–10). **e** *Pst::LUX* colonisation of tomato plants was quantified 3 days post inoculation with 4 different doses of *Pst::LUX* (*n* = 8). Letters indicate significant differences (*p* < 0.05) between groups according to a Tukey post-hoc test, conducted following a one-way ANOVA. Lower, middle and upper horizontal lines in boxplots (**c**–**e**) indicate the 1st, 2nd and 3rd quartiles; whiskers extend to the lowest and highest data points within 1.5× interquartile range below and above the 1st and 3rd quartiles.

To investigate the impact of hydroponic growth on disease susceptibility of tomato, plants (cv. Moneymaker) were grown from seed in stone wool (Fig. 1a), compost, or topsoil and spray inoculated at 19 days post sowing with a bioluminescent strain of *Pseudomonas syringae* pv. *tomato* DC3000 (*Pst::LUX*), the causal agent of bacterial speck disease. Three days post inoculation, leaves were detached, and quantification of bacterial colonisation was assessed by bioluminescence (Fig. 1b). Stone wool grown tomato sustained dramatically enhanced levels of *Pst::LUX* colonisation in leaves compared to compost or topsoil grown plants (Fig. 1c). These differences in disease susceptibility were not related to plant size as stone wool grown plants were intermediate between topsoil- and compost-grown plants (Fig. 1d). Thus, stone wool grown tomato was significantly more susceptible to foliar disease than soil or compost grown tomato.

### Microbes can induce ISR in stone wool-grown tomato

**Fig. 2:**
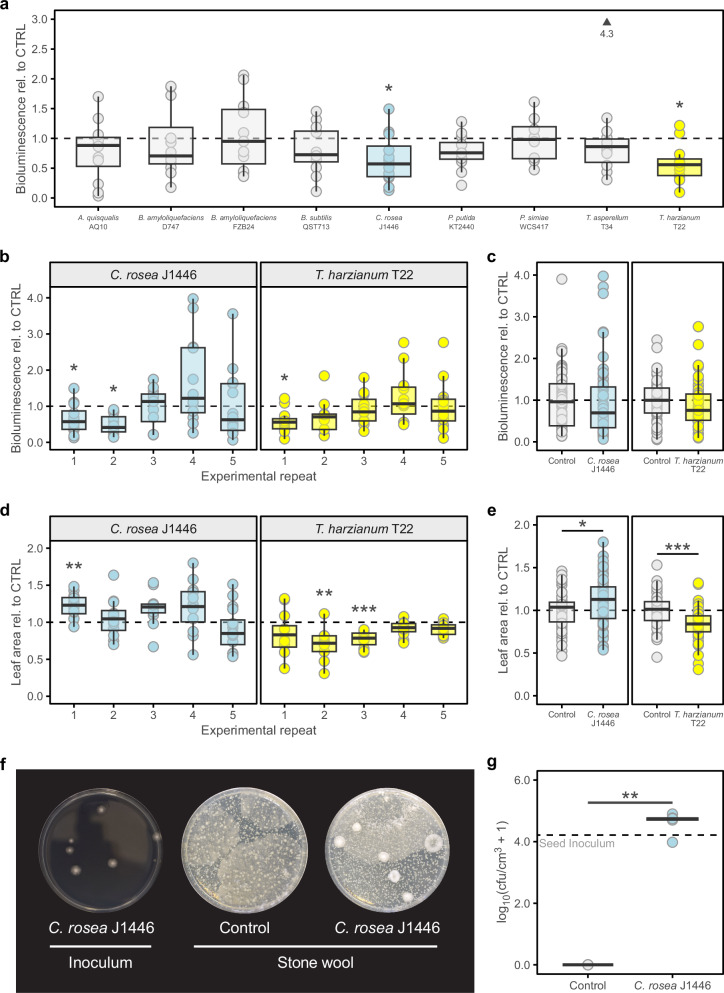
*Clonostachys rosea* J1446 suppresses disease and promotes growth in stone wool-grown tomato. *Pst::LUX* bioluminescence expressed relative to uninoculated controls (CTRL; black dashed line is control mean) for tomato (cv. MoneyMaker) treated at sowing with one of 9 independent bacterial and fungal strains in a single experiment (**a**) or treated at sowing with *C. rosea* J1446 or *Trichoderma harzianum* T22 in 5 independent experimental repeats which are shown separately (**b**) and all together (**c**). Results shown for experimental repeat 1 in (**b**) are the same as those shown in (**a**). Arrowhead in (**a**) points to datapoint outside plotting area. Points equate to the average of two leaves from a single plant (*n* = 8–20 for the individual experiments). **d**, **e** Leaf area expressed relative to mean of the uninoculated CTRLs (black dashed line) for the same plants as imaged in (**b**) and (**c**). Points equate to the average of two leaves from a single plant (*n* = 9–20 for the individual experiments). **f** Representative potato dextrose agar plates showing ×2500 dilution of the *C. rosea* J1446 inoculum added at sowing and ×50 dilution of stone wool plugs sampled 20 days post sowing, from uninoculated control and *C. rosea* J1446 treated plants, and shaken in 50 ml of sterile water. **g** Counts of *C. rosea* J1446 like colonies from stone wool plugs sampled 20 days post sowing. The black dashed line indicates the estimated *C. rosea* J1446 dosage at sowing. Asterisks indicate a significant difference between microbe and water control as determined by two-tailed *t*-test (*p < 0.05; **p < 0.01; ***p < 0.001).

Root-colonising microbes can elicit an induced systemic resistance (ISR) response in plants, which protects against a wide spectrum of pests and diseases^23^. To identify bacterial or fungal isolates mediating ISR in stone wool-grown tomato plants, we inoculated stone wool immediately post sowing with nine different individual bacterial or fungal strains, including commercially available biocontrol strains or strains that had previously been identified as being disease suppressive in soil-based studies. Resistance to *Pst::LUX* was quantified at 3 days after inoculation of ~18-day-old plants, using an inoculation density of OD_600nm_ = 0.01, which allowed for high levels of bioluminescence (Fig. 1e) without causing extreme disease symptoms that could obscure subtle ISR effects. Of the nine strains tested, *Clonostachys rosea* J1446 and *Trichoderma harzianum* T-22 significantly reduced the colonisation by *Pst:LUX* relative to control plants (Fig. 2a). To assess the robustness of this disease suppression, we conducted five independent repeats for these two strains (Fig. 2b, c). Intriguingly, we found that while *Pst:LUX* colonisation was generally reduced, it was not always statistically significant. Notably, some experiments showed a marked increase in variation by the biocontrol treatment, with some leaves exhibiting increased colonisation, whilst other showing reduced colonisation compared to the control group (Fig. 2b).

Resistance-inducing microbes also often promote growth through a variety of direct and indirect mechanisms^17^. In our trials, *C. rosea* J1446 significantly promoted growth (two-tailed *t*-test, *p* = 0.02) whereas *T. harzianum* T-22 significantly repressed growth (two-tailed *t*-test, *p* < 0.001) (Fig. 2d, e). However, as with the colonisation by *Pst:LUX*, there was variation between experiments in the extent of growth promotion and repression (Fig. 2d).

Due to the ability for *C. rosea* J1446 to elicit ISR and promote growth in stone wool-grown tomato, we focused our attention on this microbe. To understand its persistence, we sampled stone wool at 20 days post sowing and plated extracts on the general fungal media potato dextrose agar. Based on colony morphology (Fig. 2f), we concluded that *C. rosea* J1446 persisted in all samples with on average 5.1 ×10^4^ CFU per cm^3^ of stone wool and there was no cross-contamination of the controls (Fig. 2g). Thus, *C. rosea* J1446 persists in stone wool for at least several weeks, mediating both ISR and growth promotion.

### Synthetic microbial communities elicit ISR and promote plant growth

To improve the reproducibility of the plant-beneficial effects of microbial inoculants, we established small three-member synthetic communities (SynComs) that included the most promising isolate from our single-isolate assays, *C. rosea* J1446. Both SynComs consisted of two fungal isolates and one bacterial strain to increase trait diversity. The first, called “Commercial SynCom”, combined *C. rosea* J1446 (Prestop) with *Bacillus subtilis* QST713 (Serenade ASO) and *Trichoderma harzianum* T-22 (Trianum-P). Serenade is a long-established, commercially available biopesticide^24^ and Trianum-P has resistance inducing capabilities in stone wool-grown tomato (Fig. 2a, b). The second SynCom, called “Laboratory SynCom”, combined *C. rosea* J1446 with two non-commercial strains, *Clonostachys rosea* IK726^25^ and *Pseudomonas chlororaphis* PCL1391^16^. *C. rosea* IK726 has been well-studied, including extensive trialling at scale^26^. Also, it has been shown that *C. rosea* can tolerate the anti-fungal metabolites produced by *P. chlororaphis*^27^.

**Fig. 3:**
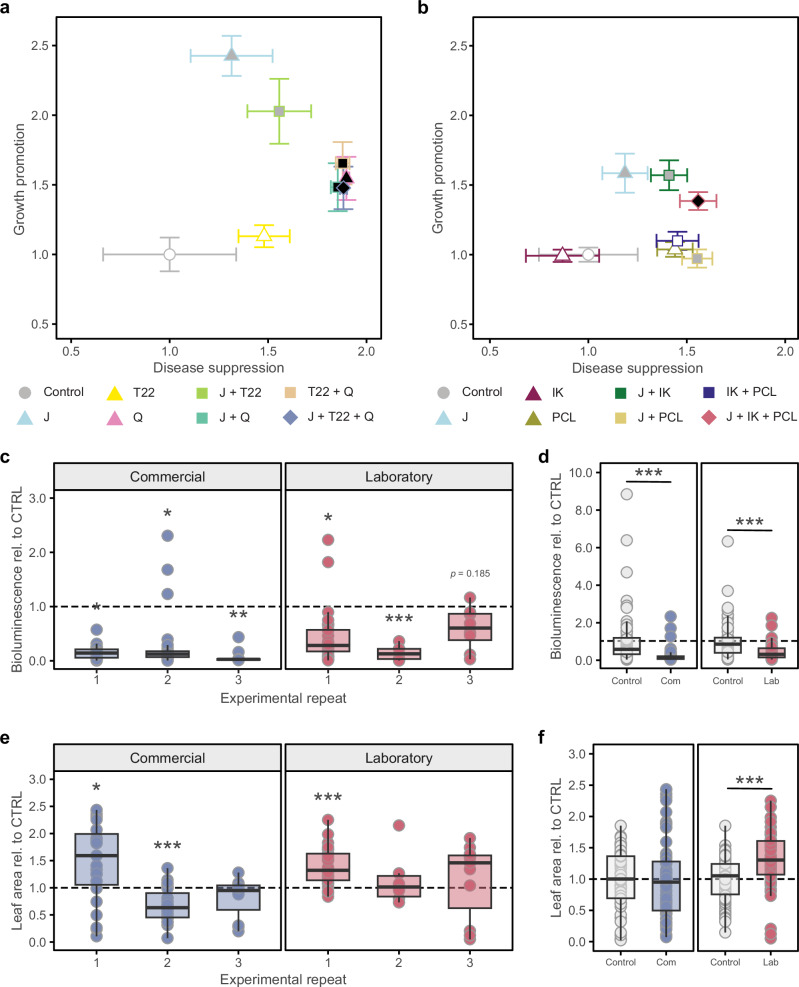
Synthetic communities (SynComs) of plant-beneficial microbes consistently suppress disease in stone wool-grown tomato. **a**, **b** Growth promotion vs disease suppression in week-old tomato (cv. MoneyMaker) seedlings for two 3 member SynCom’s compared to control and constituent members on their own or in pairs. Growth promotion is the green leaf area expressed relative to control. Disease suppression is 2 - (mean *Pst::LUX* bioluminescence expressed relative to control). Points are means (*n* = 20–30) ± SEM. Dot shape indicates number of microbes in the treatment, circle = control, triangle = 1, square = 2 and diamond = 3. Dot fill indicates whether there is a significant difference between microbe/SynCom and control for growth promotion and/or disease suppression as determined by two tailed *t*-test (white *p* > 0.05 for both parameters; dark grey *p* < 0.05 for one parameter; black *p* < 0.05 for both parameters). J, *Clonostachys rosea* J1446; T22, *Trichoderma harzianum* T22; Q, *Bacillus subtilis* QST713; IK, *Clonostachys rosea* IK726; PCL, *Pseudomonas chlororaphis* PCL1391. **c**, **d** *Pst::LUX* bioluminescence expressed relative to uninoculated controls (CTRL; black dashed line is control mean) for tomato (cv. MoneyMaker) treated at sowing with the Commercial (J + T22 + Q) or Laboratory (J + IK + PCL) SynComs in 3 independent experimental repeats, which are shown separately (**c**) and all together (**d**). Points equate to single plants (*n* = 6–29 for the individual experiments). **e**, **f** Leaf area expressed relative to mean of the uninoculated CTRLs (black dashed line) for the same plants as imaged in (**c**) and (**d**). Points equate to the leaf area of a single plant (*n* = 6–29 for the individual experiments). Asterisks indicate a significant difference between SynCom and water control as determined by two-tailed *t*-test (*p < 0.05; **p < 0.01; ***p < 0.001).

SynComs were assessed for their ability to induce resistance to disease (*Pst::LUX*) and promote growth in week-old tomato plants by comparing to a control along with single or pairs of SynCom members (Fig. 3a,b). Plants inoculated with the Commercial SynCom showed significantly reduced colonisation by *Pst::LUX* and were significantly larger than the water-treated controls (Fig. 3a). Similar benefits were provided by *B. subtilis* QST713 on its own or in combination with *T. harzianum* T-22 or *C. rosea* J1446 (Fig. 3a), suggesting that *B. subtilis* QST713 was a key driver of plant-beneficial responses. In contrast, for the Laboratory SynCom, only the complete three-member community significantly reduced colonisation by *Pst::LUX* and significantly increased plant size (Fig. 3b). Thus, the evidence from these first experiments suggests that both the Commercial and Laboratory SynComs elicit ISR and promote growth of hydroponically grown tomato.

To confirm the reproducibility of the Commercial and Laboratory SynComs benefits, we repeated the experiments but only included the SynComs and their respective controls. Both SynComs consistently suppressed *Pst:LUX* colonisation (Fig. 3c, d). This was particularly true of the commercial SynCom which strongly and significantly suppressed *Pst:LUX* colonisation in all three repeats. However, although the Laboratory SynCom did overall significantly suppress colonisation (Fig. 3d), in one of the individual repeats the suppression was not statistically significant (Fig. 3c). Regarding SynCom-induced growth promotion, the patterns were more inconsistent (Fig. 3e, f). The Laboratory SynCom significantly promoted growth when the three experiments were combined (Fig. 3f). However, the Commercial SynCom was much more inconsistent as it significantly promoted growth in one experiment and significantly repressed growth in another (Fig. 3e). Nevertheless, despite the inconsistent impacts on growth both SynComs robustly suppress disease in hydroponically grown tomato.

### Testing SynCom performance under commercial conditions

**Fig. 4:**
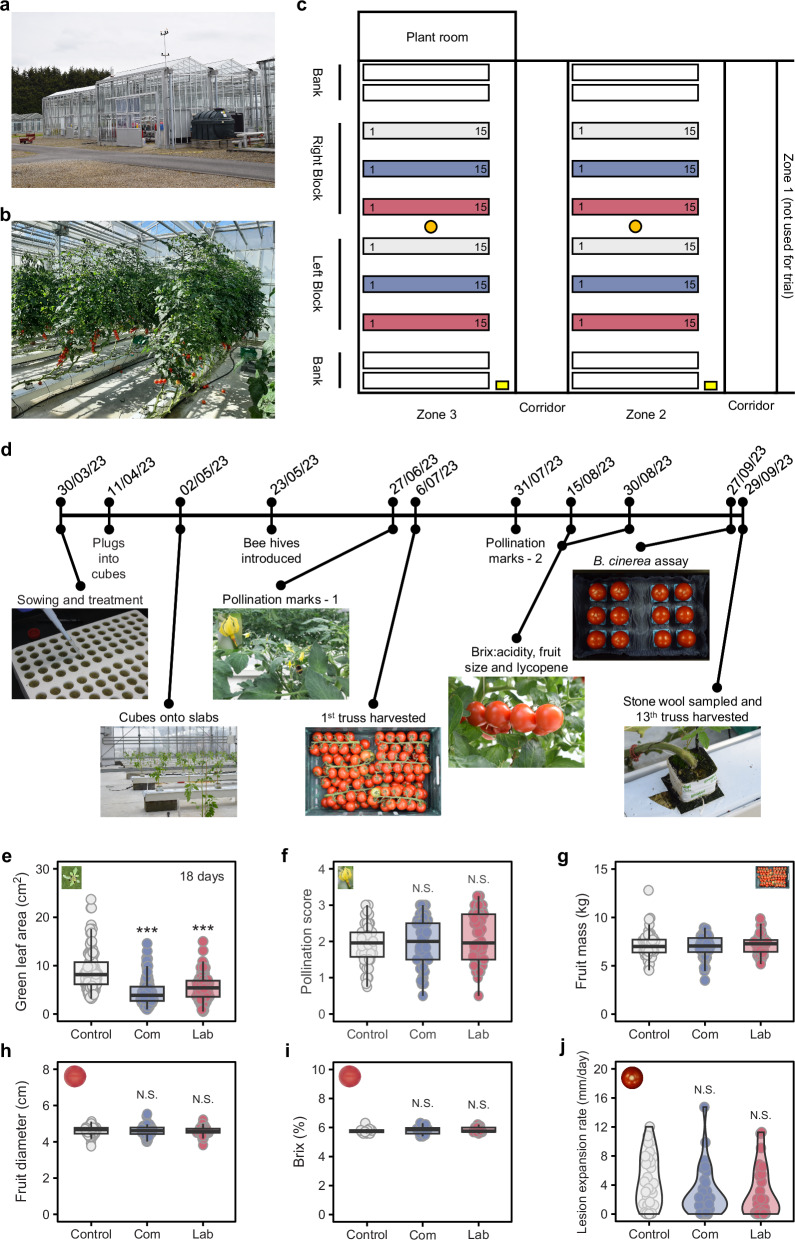
Testing laboratory developed Synthetic communities (SynComs) of plant-beneficial microbes at commercial scale. **a** Advanced glasshouse at Stockbridge Technology Centre (Selby, UK). **b** Two of twelve rows of tomato (cv. Arlinta) plants in the 6-month trial. Plants were grown in stone wool and fed nutrients through dripper lines. **c** Layout of the 6-month trial. The trial was replicated across two greenhouse compartments (Zone 2 and 3) with each containing two blocks of three rows of 15 tomato plants one from each of the three treatment groups, Control (grey), Laboratory SynCom (red) and Commercial SynCom (blue). To minimise edge effects, two guard rows were included at the edge of each zone (white) and plants 1 and 15 from each row were also regarded as guard plants. Rows drained in the direction of plant 1. Bee hives and pollen deposits are depicted by yellow boxes and orange circles, respectively. **d** Timeline of key steps in the trial. **e** Green leaf area of plants (*n* = 102–118) growing in stone wool cubes at 18 days post sowing. **f** Pollination score for plants (*n* = 50–52) growing on stone wool slabs at 89 days post sowing (27.06.23). Open flowers were assigned to one of five categories based on degree of bumble bee induced bruising of the anther cone (0 no bruising and no pollination to 4 severe bruising and heavily pollinated^62^). The pollination score is the mean per plant bruising level. **g** Fruit yield from the 12 trusses harvested between 98- and 182-days post sowing. Points are the mass of all fruit for 1 truss from all plants of a row. There was no effect of treatment on fruit yield (LMEM: Χ^2^ = 1.516, df = 2, *p* = 0.4686). **h** Average per plant diameter (*n* = 49–51) for fruit harvested from truss 6 on 138 days post sowing. **i** Percentage sugar content of fruit harvested from truss 8 on 153 days post sowing. Brix measurements were taken from the juice of 6 blended tomatoes collected from the 3 plants of one stone wool slab. Only the 3 middle slabs of every row were sampled (*n* = 12). **j** Rate of expansion of *Botrytis cinerea* necrotic lesions between 3 and 5-days post inoculation of fruit harvested from truss 11 on 181 days post sowing. Points represent the average of two fruit from individual plants (*n* = 27–29). Asterisks indicate a significant difference between SynCom and water control as determined by two-tailed *t*-test (N.S. *p.adj* > 0.05; *p.adj < 0.05; **p.adj < 0.01; ***p.adj < 0.001).

Having demonstrated the possible benefits of SynComs to tomato at early stages of development, we wanted to understand the longer-term impacts of introducing SynComs into a commercially representative hydroponic production system. Specifically, we wanted to determine the impacts of our SynComs on commercially relevant crop traits, including plant growth, fruit yield and fruit quality, in relation to the persistence of SynCom members and impacts on the hydroponic microbiome over a growing season. We therefore undertook a commercial demonstrator trial at the Stockbridge Technology Centre (Selby, UK), in an advanced greenhouse (Fig. 4a) using commercial hydroponic systems and horticultural practices (Fig. 4b). This trial used a commercial red cocktail tomato variety, Arlinta, which grew much more rapidly and larger than Moneymaker (Supplementary Fig. 1a). Preliminary testing under laboratory conditions had revealed that Arlinta has strong innate resistance to *P. syringae* pv. *tomato* DC3000 (Supplementary Fig. 1b); accordingly, the trial did not involve additional disease testing with this pathogen. The trial compared plants inoculated with either the Commercial or Laboratory SynComs to uninoculated controls (Fig. 4c). SynComs were applied once at sowing, and then the trial was run for 6 months during which time multiple growth and development parameters were assessed (Fig. 4d).

Both SynCom treatments slightly inhibited seed germination, with only 33.3 and 40% of Commercial and Laboratory, respectively, having germinated by 7 days post sowing in comparison to 49.3% for the control (Supplementary Table 2). At 18 days post sowing, after seedlings had been transferred to stone wool cubes, SynCom-treated plants were significantly smaller (Fig. 4e). This difference persisted after plants had been transferred to stone wool slabs, with SynCom treatment plants being significantly shorter than controls until at least 71 days post sowing (Supplementary Table 2). Interestingly, this small reduction in early plant growth did not result in long-lasting negative impacts on other traits.

Pollination by bumble bees increases yield and quality of tomato fruit and therefore bumble bee hives are used in commercial tomato glasshouses^28^. To address the effects of the SynComs on pollination, we assessed pollination at two time points by recording anther cone damage of open flowers. In both cases, despite interzonal variation, neither SynCom had a significant effect on pollination (Fig. 4f and Supplementary Table 2).

Fruit yield was recorded per row (Fig. 4c) for all trusses from 98 to 182 days post sowing, by which time 12 trusses had developed per plant. In total, more than a metric tonne of tomatoes were harvested, with 343.87, 344.56 and 335.96 kg for the control, Laboratory and Commercial treatments, respectively (Supplementary Table 2). No differences in fruit yield were observed (Fig. 4g and Supplementary Table 2). For four trusses (6, 8, 9 and 11), yield data per plant were recorded, also revealing no significant impacts of either SynCom treatment (Supplementary Table 2).

Consumer appreciation of tomato fruit is influenced by size, colour, textual behaviour and flavour^29–31^. The major determinants of tomato fruit flavour are sugar content (mainly glucose and fructose), acids (primarily citrate, malate and ascorbate) and their ratio^32–34^. Measurements of fruit ripeness, diameter, brix (measure of fruit sugar content), acidity, and brix:acidity ratio were examined for truss 6 (138 days post sowing; Fig. 4h, Supplementary Fig. 2 and Supplementary Table 2). Brix, acidity and brix:acidity ratio were measured again on truss 8 (153 days; Fig. 4i and Supplementary Table 2). Textural parameters (rupture force, deformation to rupture and firmness) were measured on Truss 11 only (Supplementary Table 2). No significant differences between control plants and plants inoculated with the Laboratory SynCom were observed for any of the parameters tested at any timepoint (Supplementary Table 2). For plants inoculated with the commercial SynCom, all parameters were the same as the control except for a small reduction in normalised ripeness (1.08) for the fruit of truss 6 (Supplementary Table 2). These fruits would still be considered ‘red’ for commercial purposes and could have a longer shelf life, however this requires further testing including post-harvest storage experiments.

Substantial losses can be incurred due to post-harvest diseases^35^. Therefore, we assessed the post-harvest susceptibility of fruit to the problematic fungal pathogen *Botrytis cinerea*^36^. At 3 days post inoculation (dpi), the average lesion diameter on fruit from Laboratory and Commercial SynCom-treated plants was reduced in comparison to the control fruit. The subsequent lesion expansion rate was reduced from an average 4.67 mm day^−1^ for control fruit to 3.04 and 3.15 mm day^−1^ for fruit from Laboratory and Commercial SynCom-treated plants, respectively (Fig. 4j, Supplementary Table 2). However, the high variation in disease development between individual fruits meant these differences were not statistically significant (Control Vs Laboratory SynCom, *t*-test, *p.adj* = 0.096; Control Vs Commercial SynCom, *t*-test *p.adj* = 0.100; Fig. 4j, Supplementary Table 2).

In summary, application of SynComs under commercial-like hydroponic growth conditions of tomato does not have a deleterious impact on final marketable yield despite reducing germination efficiency and early vegetative growth.

### Profiling the stone wool hydroponic microbiome

To determine whether the SynCom members persisted and their long-term impact on the stone wool microbiome, samples were collected at the end of the 6-month trial from three locations: i) on top of the stone wool cube next to the tomato stem base (T), ii) in the stone wool slab next to the base of the stone wool cube (Slab) and iii) in the middle of the stone wool slab (Mid_slab; Supplementary Fig. 3). Amplicon sequencing of bacterial 16S rRNA and fungal ITS gene regions was performed on DNA extracted from the stone wool samples.

**Fig. 5:**
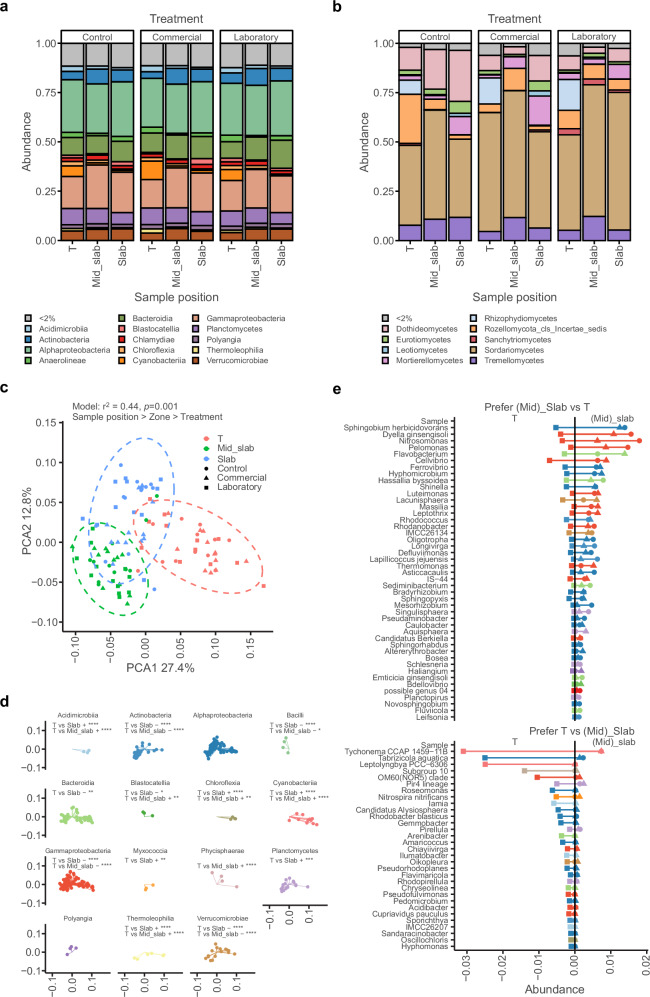
Tomato shapes stone wool microbiome. Relative abundances of bacteria (**a**) and fungi (**b**) at the Class taxonomic level. Classes with a relative abundance less than 2% are grouped together. **c** Principal component analysis (PCA) of samples using the weighted Unifrac distance metric. Ellipses show confidence levels of 0.95. **d** shows the contributions to the PCA of individual taxa, coloured by Class, that differ significantly between samples taken from the top of the stone wool cube next to the tomato stem base (T) and slab or mid_slab samples. Classes with less than 2 members have been omitted for clarity. Each panel shows whether the Class as a whole favours T (+) or (Mid_)slab samples (−). Significance is shown as *p < 0.05, **p < 0.01, ***p < 0.001, ****p < 0.0001). **e** Shows the relative abundance of ASVs that show preferential growth in T samples or (Mid_) slab samples. Only ASVs with a relative abundance >0.1%, which are significantly different in both Slab and Mid_slab samples have been plotted. Square T, Triangle Slab, Circle Mid_slab.

Absolute quantification of bacterial taxa by comparison with spike-in controls showed that bacterial numbers were high at the end of the trial (1.93–6.84 × 10^9^ cells g^−1^ substrate; Supplementary Fig. 4a, b) but also comparable to previous studies profiling stone wool and tomato roots^7^. Fungal reads were much lower, but absolute quantification was complicated as amplification of plant and algal sequences consumed a substantial proportion of the sequencing reads. Bacteria were most abundant in samples collected next to the tomato stem (Supplementary Fig. 4b). The stone wool was not covered by plastic wrapping in these areas and the substrate was noticeably green (Supplementary Fig. 3c). Alpha and gamma proteobacteria were the most abundant bacterial classes (Fig. 5a and Supplementary Fig. 4c), whereas the most abundant fungal class was Sordariomycetes of the Ascomycota (Fig. 5b).

Principal Component Analysis (PCA) of the 16S rRNA data showed clear clustering with sample position contributing most to the separation, followed by greenhouse zone, then SynCom treatment (Fig. 5c). We explored the contribution of individual ASVs to this clustering and identified classes for which sample location had a statistically significant effect (Fig. 5d). Of the classes identified, Acidimicrobiia, Chloroflexia, Cyanobacteriia and Thermoleophilia showed a strong preference for location T, whilst Actinobacteria, Bacilli, Gammaproteobacteria, Verrucomicrobiae showed a preference for Slab and Mid-slab locations (Fig. 5d). To explore this preference for T or (Mid)_slab in more detail, we identified ASVs with a relative abundance of >0.1% that were significantly different to T in both Slab and Mid_Slab (Fig. 5e). The eight ASVs with strongest preference for (Mid)_slab over T were all Gram-negative, rod-shaped, aerobic bacteria commonly found in soil and water samples (Fig. 5d), such as *Sphingobium herbicidovorans* and bacteria of the ammonia-oxidising chemolithoautotrophic genera, *Nitrosomonas*. Those ASVs with the strongest significant preference for T over (Mid)_slab also included Gram-negative, rod-shaped, aerobic bacteria such as *Tabrizicola aquatica*. However, generally, the ASVs with strongest preference for T were less well-defined Cyanobacteria or bacteria previously isolated from aquatic environments (Fig. 5e). In summary, clear differences in the bacterial community were observed between different stone wool sampling positions.

### Assessing SynCom microbe persistence

**Fig. 6:**
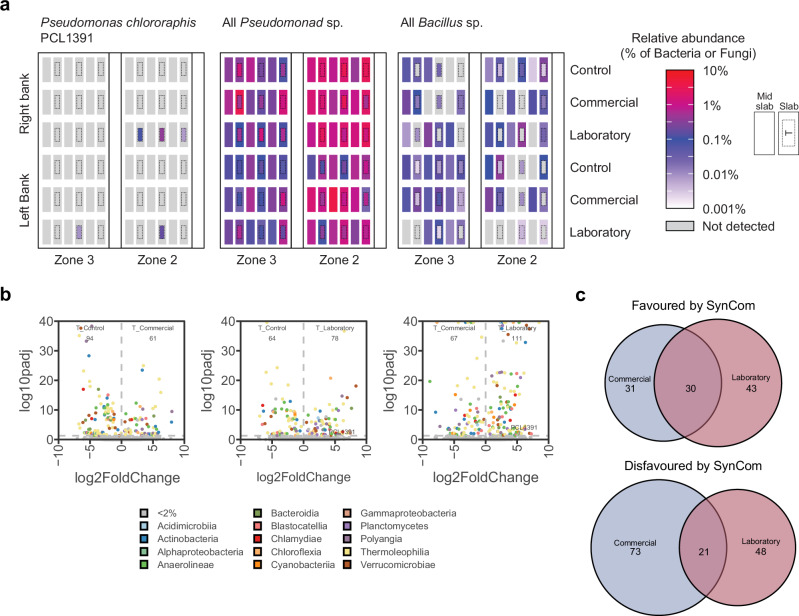
*Pseudomonas chlororaphis* PCL1391 persisted in stone wool for six months and fits general trend of *Pseudomonads* excelling in tomato hydroponic systems. **a** The relative abundance of the PGPR *Pseudomonas chlororaphis* PCL1391, all *Pseudomonad* sp. and all *Bacillus* sp. in samples taken from the greenhouse. **b** Coloured points represent ASVs which differ significantly (*p.adj* < 0.05) in relative abundance between indicated treatment groups at the T sampling position. Colours indicate the ASVs taxonomic assignment at the Class level. **c** ASVs significantly favoured in the T stone wool sampling position of the commercial and/or laboratory SynCom treatments.

As SynComs were added only once, when seeds were sown, we wanted to determine if SynCom members had persisted within the complex hydroponic microbiomes throughout the cultivation period and until the end of the 6-month trial. SynCom members were identified on the basis of 100% sequence identity (as confirmed by sequencing the input SynCom community). Where detected, they were only present associated with plants to which the specific SynCom had been added, and not in other treatments. This confirms i) that there was no cross-contamination between treatments and ii) that other hydroponic microbiome community members did not share the 16S/ITS sequences. Of the bacteria, *Pseudomonas chlororaphis* PCL1391 was detectable in 5 out of the 36 samples taken from Laboratory SynCom treatment rows with a relative abundance between 0.007% and 0.35% (Fig. 6a). This ASV was only detected in samples from the T position (Supplementary Fig. 3), which made up 12 of the 36 samples taken from Laboratory SynCom treatment rows (Fig. 6a). The Commercial SynCom member *Bacillus subtilis* QST713 was not detected in any samples. To determine if the retention of *P. chlororaphis* PCL1391 and loss of *B. subtilis* QST713 was related to the overall prevalence of these genera, the relative abundance of all *Pseudomonad* and *Bacillus* ASVs were plotted (Fig. 6a). There was a high relative abundance of *Pseudomonads* across both zones and all sampling positions, but particularly the T sampling position (Fig. 6a). In contrast, *Bacillus* ASVs had a much lower relative abundance in general and particularly in the T sampling position (Fig. 6a). Thus, retention of *P. chlororaphis* PCL1391 and loss of *B. subtilis* QST713 aligns with the abundance of their genera in tomato stone wool.

Of the fungal SynCom members, only one sample contained an ASV that matched *Clonostachys rosea* J1446/IK726 (relative abundance 2.6% in sample 17 – a T position, Laboratory SynCom sample) and none matched *Trichoderma harzianum* T22. However, these results must be caveated by the fact that the number of fungal reads obtained was low due to the amplification of tomato sequences.

In summary, of all SynCom members applied, only *Pseudomonas chlororaphis* PCL1391 persisted consistently throughout the duration of the experiment in multiple samples. By contrast, *Bacillus subtilis* QST713 did not persist, and fungal members were only sporadically detected in one or two samples.

### SynComs introduced at sowing have lasting impacts on the stone wool microbiome

Although the presence of individual SynCom members at the end of the trial was low, we explored whether the early presence of SynComs had long-lasting impacts on the community structure of the hydroponic microbiome. We identified ASVs that differed significantly in relative abundance between treatment groups at the T sampling position (Fig. 6b). In comparison to the control, both SynComs favoured and disfavoured 61–94 ASVs (Fig. 6c). Furthermore, 30 and 21 favoured and disfavoured ASVs, respectively, overlapped between the two SynComs (Fig. 6c). Thus, although the SynCom members did not persist at high relative abundance, they did have persistent founder effects on the hydroponic microbiome of tomato.

## Discussion

With the human population growing rapidly and forecast to continue growing for the coming fifty to sixty years^37^, increased pressure is being placed on land, soil and water resources^38^. The Food and Agriculture Organization of the United Nations (FAO) highlighted in a recent report how hydroponic production systems, which efficiently use space and water offer a solution, particularly with the growing importance of urban and peri-urban agriculture in food production^38^. In this study, we provided, to our knowledge, the first evidence for how SynComs of plant beneficial microbes could play an important role in further improving hydroponic production systems.

Stone wool is a widely used and proven substrate for commercial hydroponic production, particularly for crops such as tomato^39^. In comparison to soil, stone wool provides a very different physical, chemical as well as biological growing environment. Soil has a complex microbiome, which is present from the moment of germination, whereas stone wool starts as a sterile environment and is increasingly colonised across the crop growing cycle^7,40,41^. The soil microbiome contains a range of organisms, some of which can be beneficial by increasing plant growth and/or suppressing plant disease^42,43^. There has been work over several decades to introduce beneficial microbes isolated from soil to stone wool-based growing systems^8,14^. Such introductions could potentially provide a sustainable solution for disease protection while also reducing energy and nutrient input requirements by promoting plant growth. We demonstrated here how single strains can induce systemic resistance against disease and promote growth of tomato growing in stone wool (Fig. 2a). However, we also demonstrated a common problem in that the benefits provided by single strains are often inconsistent (Fig. 2b–e). We proposed that a solution to this inconsistency could be introducing multiple strains and taking advantages of the benefits provided by diverse communities.

There is evidence from across kingdoms of how highly diverse communities exhibit enhanced robustness to perturbations and retain functions more effectively than communities containing only one or two species^44–46^. The classic example was provided by Tilman and Downing who demonstrated that more diverse grassland communities were more resistant and resilient to perturbation in the form of severe drought^47^. Intriguingly, it was subsequently shown that the diversity of soil microbes in the form of arbuscular mycorrhizal fungi can drive the biodiversity of plant communities and in turn ecosystem function^48^. Based on this literature we hypothesised that small communities of beneficial microbes could provide more consistent resistance and growth benefits to plants than single strains. However, there has been only limited research on the use of communities of beneficial microbes in hydroponic horticulture^8^. Here, we combined the most promising individual strains with microbes previously shown in soil environments to elicit ISR and/or promote growth, to create two small synthetic communities (SynComs). In both cases the SynComs induced resistance to disease and potentially promoted growth, at a level as great or greater than any of the individual isolates (Fig. 3). It is interesting to note that there does not appear to be any synergistic effects on growth and disease resistance with the two SynComs. Instead, disease suppression and growth promotion appears to be strongly driven by individual isolates with minor additive effects of the others. Nevertheless, the SynComs still have a major advantage when it comes to consistency. If for any reason individual isolates drop out, the others are still there to provide a degree of redundancy. Furthermore, in this study, we only assessed the enhanced resistance against one pathogen and the different microbes with different traits could provide benefits against other pathogens. Future work should explore this while also assessing whether the SynComs enhance robustness to other perturbations common in glasshouse-based hydroponic tomato growing, such as heat spikes on sunny days. Adding additional microbes to the SynComs and looking for synergistic interactions could also be considered.

Juveniles are often more susceptible to disease than adults and in plants this can be explained by age-dependent resistance^49,50^. We demonstrated that treatment of stone wool with two three-member SynComs could protect vulnerable juvenile hydroponically grown tomato plants against disease (Fig. 3). Crucially, we showed this protection was systemic as we found tomato plants growing in SynCom-treated stone wool displayed enhanced resistance to a foliar pathogen, specifically *Pseudomonas syringae* pv. *tomato* DC3000. This pathogen is the causal agent of bacterial speck disease and is a major problem for commercial hydroponic tomato production^12^. Thus, the results generated in this study are of commercial relevance.

Although the disease protective abilities of the SynComs are important, they would be of little use in a commercial setting if they had deleterious effects on fruit yield and fruit quality when disease pressure was low. Therefore, we extensively profiled the performance of tomato plants growing in SynCom-treated stone wool under simulated commercial conditions. Plants growing in SynCom-treated stone wool were on average 40–50% smaller in terms of green leaf area (18 days post sowing) and reduced in height by on average 3–8% (71 days post sowing) during the early phases of growth (Fig. 4e). In crops with a short cropping cycle and where the yield is green leaf tissue such as lettuce, this SynCom-induced growth repression could be a problem. However, in tomato, which has a much longer cropping cycle and where the yield is in the form of fruit, the growth repression may not be problematical in a commercial setting. Shorter plants would be easier from a fruit harvesting perspective. Furthermore, we provided clear evidence that the SynComs had no long-lasting effect on fruit yield and quality despite the early growth repression (Fig. 4). Ensuring a high fruit yield requires successful pollination, and this function is commonly performed in commercial tomato greenhouses by introduced bumble bees^28^, which can be sensitive to defence-related changes to floral phenotypes. We showed that SynCom treatments had no negative effects on bumble bee visitation and pollination (Fig. 4f). In turn, fruit yield was almost identical across the three treatment groups (Fig. 4g). For fruit quality we assessed fruit size, colour, textural behaviour and flavour determinants, as these influence a consumer’s appreciation of a tomato fruit^29–31^. All parameters measured were unchanged by SynCom treatments, except for a small delay in fruit ripening for the commercial SynCom treatment. Thus, the SynComs formulated in this study can protect vulnerable young plants without having negative long-term impacts on fruit yield and fruit quality parameters. However, although we extensively profiled different parameters linked to fruit taste, the proof is in the eating and future work should conduct taste tests with expert panels of individuals trained in sensory evaluation.

An issue that is also pertinent to commercial tomato production is shelf-life and susceptibility to post-harvest diseases such as *Botrytis cinerea*, the causal agent of grey mould^36^. We found that the lesion expansion rate of *B. cinerea* on fruit of SynCom plants was on average 34% slower than on control fruit (Fig. 4j). However, due to high inter-fruit variability this resistance was not significant (Supplementary Table 2) and was weaker than the protection displayed in young plants against bacterial speck disease. This could be a result of ISR wearing off or may be linked to the pathogen and their lifestyle. Future work should explore the long-term effects on resistance against a range of biotic threats.

Previous studies have developed SynComs, which have been shown to protect soil-grown crops, including tomato, against (a)biotic stress. For example, Schmitz and colleagues identified a five-member bacterial SynCom capable of protecting tomato plants against high salt stress^51^. They identified by amplicon sequencing the presence of several of the SynCom isolates in the substrate or associated with the plant at the end of the experiment. While interesting, the plants were only three weeks old. Studies with SynComs generally lack long-term evaluation of SynCom presence under commercially relevant growing conditions^22^. Here, we evaluated at the end of our 6-month growing trial whether any of the introduced SynCom microbes had persisted. We found that although *C. rosea* J1446 persisted for 20 days in stone wool under laboratory conditions (Fig. 2f, g), none of the introduced SynCom fungal isolates consistently persisted across the 6-month commercial trial. However, we found that one SynCom bacterium, *Pseudomonas chlororaphis* PCL1391, persisted in multiple independent samples from close to the tomato stem and the site of inoculation (Fig. 6a). In contrast, the other SynCom bacterial isolate, *Bacillus subtilis* QST713, did not persist in any samples. The trend exhibited by these two SynCom microbes matched a more general pattern at a genera level. Pseudomonads were abundant, particularly around plants, whereas ASVs of the *Bacillus* genera were found at much lower abundance and this was particularly true for the samples closest to plants (Fig. 6a). Unlike in soil, where the microbiome has been profiled extensively, previous studies looking at the hydroponic microbiome have been more limited. Nevertheless, a recent study profiled the bacterial community in stone wool from commercial tomato greenhouses^41^. In samples gathered from close to the tomato stem, and therefore comparable to this study’s T sampling position (Supplementary Fig. 3a, c), they found that *Pseudomonas* was one of the top ten most abundant bacterial genera, whereas *Bacillus* was not^41^. However, recent work has shown that *B. subtilis* can rapidly evolve to become a better root coloniser of *Arabidopsis thaliana* growing hydroponically^52^. Thus, although *B. subtilis* QST713 did not persist in our study and isolates of the *Bacillus* genera more generally don’t appear to be abundant close to stone wool-grown tomato plants, there is evidence that *Bacillus* sp. can adapt to thrive in hydroponics.

In addition to assessing SynCom microbe persistence, we also looked at whether there were any impacts on the wider bacterial microbiome 6 months after the SynComs were introduced. Strikingly, we identified tens of ASVs which were significantly favoured or disfavoured in plant-associated stone wool samples from the SynCom treatments when compared to the control (Fig. 6c). Previously, Lee et al. found that the microbiome diversity and community structure in windowfarm hydroponic systems growing lettuce with clay pellet supporting media, was substantially altered by the introduction of *Pseudomonas chlororaphis* ATCC 9446^53^. However, unlike our study, where the microbiome was profiled 6 months after the introduction of the SynComs at sowing, the lettuce trial only lasted 10 weeks and *P. chlororaphis* was inoculated around the roots three times. Our results provide evidence that introduction of beneficial microbes and SynComs into hydroponic systems can have founder effects and could enable benefits for the plant to be provided even when there is low persistence of the introduced microbes themselves. In our trial simulating commercial conditions, we did not find any yield benefits, however the disease pressure was low. In future work it would be interesting to test whether yield is protected if plants experience disease pressure throughout the growing season.

In conclusion, we identified small three-member SynComs which, when introduced to stone wool-based hydroponic systems at sowing, are capable of suppressing disease in young tomato plants; do not have long-lasting adverse effects on fruit yield or quality, despite reducing germination and early vegetative growth under certain conditions; and even if they don’t all persist, have foundational effects on the long-term hydroponic microbiome. Future studies should use meta-genomics/transcriptomics to explore what enables microbes to effectively interact with their plant host, interact with other community members and survive in the hydroponic microbiome. Based on this work predictions can be made on what organisms should function best together to promote growth and disease resistance of tomato in stone wool-based hydroponic systems. This will enable further optimisation of the SynComs and in turn provide a tool for sustainable hydroponic tomato production in the long term.

## Methods

### Plant materials and growth conditions for laboratory experiments

Experiments utilised the tomato (*Solanum lycopersicum*) varieties Moneymaker (Thompson & Morgan) and Arlinta (Enza Zaden). Seeds were sterilised by vortexing in 100% ethanol for 30 s and bleach (4.37% Sodium Hypochlorite) for 4 min before being washed with sterile deionised water (dH_2_O). Seeds were sown just below the surface of 2.5 cm stone wool propagation cubes (Grodan) soaked in H_2_O and placed to germinate at 90–100% relative humidity (RH) in sealed propagator trays. Unless plants were being used for seedling experiments, propagator cubes were transferred to 10 cm Delta stone wool blocks (Grodan) at 6 days post sowing. Stone wool blocks were pre-soaked in 5 mL L^−1^ of PlantStart propagation feed (Vitalink) adjusted to pH 6 with KOH. Plants were subsequently delivered 40 mL per day of the same nutrient solution via individual dipper lines. Dripper systems were cleaned between experiments by soaking in 10 g L^−1^ Rely+On disinfectant (Virkon, CLE1554) and rinsing thoroughly with H_2_O.

When comparisons were made between plants grown in stone wool, topsoil (East Riding Horticulture Ltd) or F2 + S compost (Levington Advance), nutrient solution or water was added manually. To mimic the stone wool propagation system, seeds were sown in topsoil or compost plugs of the same volume as the 2.5 cm stone wool propagation cubes. At 6 days post sowing the soil plugs were transferred to pots containing the same volume of topsoil or compost as the stone wool blocks. Environmental conditions for all laboratory experiments were as follows: 16:8 h (day:night), 25 °C, 60% RH and 200 μE m^−2^ s^−1^.

### Microbial materials

We used commercial formulations of *Bacillus subtilis* QST713 (Serenade ASO, Bayer), *Trichoderma harzianum* T-22 (Trianum-P, Koppert), *Clonostachys rosea* J1446 (Prestop, Lallemand), *Bacillus amyloliquefaciens* D747 (Amylo-X, Certis Belchim), *Ampelomyces quisqualis* AQ10 (AQ10, Biogard), *Trichoderma asperellum* T34 (T34 biocontrol, IQV Agro International) and *Bacillus amyloliquefaciens* FZB24 (Taegro, Syngenta). *Pseudomonas chlororaphis* PCL1391, kindly provided by Prof. Arthur Ram (Leiden University, The Netherlands), green fluorescent protein (GFP) tagged *Pseudomonas putida* KT2440 derivative strain FBC004^54^, yellow fluorescent protein (YFP) tagged *Pseudomonas simiae* WCS417r^55,56^, kindly provided by Prof. Corne Pieterse (Utrecht University, The Netherlands), and *Pseudomonas syringae* pv. *tomato* DC3000 *luxCDABE* (*Pst::LUX*)^57^, were stored as glycerol stocks at −80 °C. *Clonostachys rosea* IK726, kindly provided by Prof. Dan Funck Jensen (Swedish University of Agricultural Sciences, Sweden), and *Botrytis cinerea* B05.10, kindly provided by Prof. Jan van Kan (Wageningen University & Research, The Netherlands), were continuously cultured on potato dextrose agar (PDA; Oxoid, CM0139) and malt extract agar (Oxoid, CM0059), respectively, in the dark and at 15–25 °C.

### SynCom preparation

The “Laboratory SynCom”, which consisted of *C. rosea* J1446, *C. rosea* IK726 and *P. chloraphis* PCL1391, was produced by mixing equal volumes of 18.75 mg mL^−1^ Prestop (*C. rosea* J1446), a suspension of 1–3 × 10^7^ conidia mL^−1^ of *C. rosea* IK726 and a suspension of *P. chloraphis* PCL1391 OD_660nm_ = 3. Prestop was stored at −20 °C after the packet was opened for the first time and was prepared in sterile dH_2_O. *P. chloraphis* PCL1391 glycerol stocks were thawed then cultured for 17–20 h at 28 °C and 200 rpm in King’s B (KB) liquid media^58^. Cells were collected by centrifugation at 4000 × *g* for 5 min, washed with sterile dH_2_O and resuspended in sterile dH_2_O. *C. rosea* IK726 was cultured on PDA plates for 12 days. Conidia were washed from the surface of plates with sterile dH_2_O, passed through Miracloth (Millipore) to remove mycelium, counted using a haemocytometer and diluted to the required concentration in sterile dH_2_O.

The “Commercial SynCom”, which consisted of *C. rosea* J1446, *T. harzianum* T-22 and *B. subtilis* QST713, was created by mixing equal volumes of 18.75 mg mL^−1^ Prestop (*C. rosea* J1446), 2.81 mg mL^−1^ Trianum-P (*T. harzianum* T-22) and 9% Serenade ASO (*B. subtilis* QST713). The Prestop suspension was prepared as for the Laboratory SynCom. Serenade was stored at room temperature and prepared by diluting with sterile dH_2_O. Trianum-P was stored at 4–8 °C and suspended in sterile dH_2_O at the required concentration.

SynComs containing pairs of microbes were prepared by replacing one of the microbes with sterile water.

### Preparation of individual microbe inoculums

*P. simiae WCS417r* was cultured from glycerol stocks for 24 h at 28 °C on KB agar plates supplemented with rifampicin (50 µg mL^−1^) and tetracycline (10 µg mL^−1^). Bacteria were harvested by adding 10 mL of 10 mM MgSO_4_ to the plate surface, gently scraping bacteria into suspension, centrifuging at 3000 rpm for 3 min, washing with 10 mM MgSO_4_, centrifuging at 3000 rpm for 3 min and finally resuspending in 10 mM MgSO_4_ at an OD_600nm_ = 1.

*P. putida* KT2440 was cultured from glycerol stocks for 24 h at 28 °C and 150 rpm in liquid M9 minimal salt medium supplemented with glucose (0.1%) and chloramphenicol (25 µg mL^−1^). Cells were collected by centrifugation at 4000 × *g* for 5 min, washed once with sterile dH_2_O and finally resuspended in sterile dH_2_O at an OD_600nm_ = 1.

Microbes in commercial formulations were prepared by suspending or diluting, depending on formulation, in sterile dH_2_O resulting in the following final concentrations: 0.07 mg mL^−1^ Amylo-X, 0.04 mg mL^−1^ AQ10, 6.25 mg mL^−1^ Prestop, 3% Serenade, 50 mg mL^−1^ Taegro, 0.94 mg mL^−1^ Trianum-P and 0.18 mg mL^−1^ T34 Biocontrol.

### Inoculation of stone wool with SynComs or individual microbes

In all experiments, SynComs or individual microbes were inoculated onto seeds immediately post sowing by pipetting 1 mL of inoculum or dH_2_O (control) across the surface of each 2.5 cm stone wool propagation cube. For *P. simiae* WCS417r the control solution was 10 mM MgSO_4._ Following inoculation, doses of all microbes were confirmed by serial dilution and plating on solid media.

### Pathogen bioassays

*Pst::LUX* was cultured from glycerol stocks overnight at 28 °C and 200 rpm in KB liquid medium supplemented with kanamycin (50 µg mL^−1^) and rifampicin (50 µg mL^−1^). Cells were collected by centrifugation at 4000 × *g* for 3 min, washed once with 10 mM MgSO_4_ and resuspended in 10 mM MgSO_4_. Unless otherwise specified, bacterial suspensions were adjusted with 10 mM MgSO_4_ to a final concentration of OD_600nm_ = 0.01 for 17–19 day old plants or OD_600nm_ = 0.1 for 7-day old plants. All suspensions were supplemented with 0.015% (v/v) Silwet L-77 (LEHLE SEEDS, VIS-30) as a surfactant. Plants which had been held at high humidity for 12 h, were spray inoculated with *Pst::LUX* suspensions and then returned to high humidity.

*Pst::LUX* colonisation of leaves was assessed 2–3 days post inoculation by quantifying bioluminescence using a protocol adapted from a previous study^59^. For plants inoculated 17–19 days post sowing, the first two true leaves were detached and imaged, whereas plants inoculated at 7 days old were imaged without being removed from their stone wool plugs. Plants were dark adapted for 8 min, then bioluminescence was captured using a G:BOX Chemi XRQ chemiluminescence imager (Syngene) for 6 min (7-day old plants) or 4 min (17–19 day old plants). Images of leaf area were then captured by illumination from below. Images (16-bit TIFF) were analysed using ImageJ^60^. Leaf area was determined from the brightfield images following thresholding (“Auto Local Threshold” function, Method = Phansalkar). For bioluminescent images, the background was determined as the mean +3 SD of 5 random regions where no plant material was present, then subtracted. The integrated bioluminescent signal per leaf area was then calculated.

The *B. cinerea* fruit post-harvest resistance assay was conducted using a protocol adapted from a previous study^61^. Briefly, spores were collected from the surface of a malt agar plate incubated at 15–25 °C for 4 weeks and exposed to day light for 48 h at 4 days post sub-culturing. Potato dextrose broth (PDB; Oxoid, CM0962) at 10 mg mL^−1^ was gently agitated across the surface of the plate and then passed through Miracloth (Millipore, 475855) to remove any mycelium. Spore suspension was adjusted to 9 ×10^4^ spores mL^−1^ with 10 mg mL^−1^ PDB and then incubated at 25 °C for 2 h. Tomatoes, harvested the previous day and stored at room temperature overnight, were placed on plastic weigh boats on water-soaked tissue paper in propagator trays. A 2 mm diameter puncture wound was made on the tip of each tomato fruit using a needle, then 5 µL of spore suspension was added. Propagator trays were incubated at 25 °C in darkness. Lesion diameters were measured 3–5 days post inoculation.

### Quantifying *Clonostachys rosea* J1446 in stone wool

*C. rosea* J1446 persistence in stone wool propagation cubes containing 20-day-old plants was assessed by cutting the blocks into pieces, shaking for 1 h at 250 rpm in sterile dH_2_O and then vigorously vortexing for 10 s. Serial dilutions were plated on PDA, incubated at 18 °C for 3 days and then *C. rosea* J1446 colonies were counted (identified on the basis of their distinctive morphology).

### Commercial demonstration greenhouse trial

The commercial demonstration greenhouse trial was conducted at Stockbridge Technology Centre (Selby, United Kingdom). Seeds of the commercial cocktail tomato variety Arlinta (Enza Zaden) were placed on H_2_O-soaked 2 cm diameter stone wool starter plugs in polystyrene trays (Grodan) and covered in wetted vermiculite. At 8 days after sowing when seedlings had begun to emerge, a low electrical conductivity (EC) of tomato-specific nutrient feed was provided to the plants. Starter plugs were transplanted to 10 cm Delta stone wool blocks (Grodan) 12–14 days post sowing and the EC of the feed was gradually increased. During the plug and block growing stages, plants were grown in a propagation greenhouse with fluctuating temperatures (minimum = 17.1 °C, maximum = 26.4 °C, average = 19.8 °C), relative humidity (minimum = 30%, maximum = 87.2%, average = 65.5%) and radiation sum per day (minimum = 344 J cm^−2^, maximum = 1938 J cm^−2^, average = 1142 J cm^−2^).

At 33 days post-sowing, blocks were placed onto Grotop master stone wool slabs (Grodan), which were saturated in tomato feed (Supplementary Table 1), in an advanced greenhouse. Two compartments in the greenhouse were used, with 10 rows of 5 slabs in each compartment (Fig. 4c). The outer two rows at each end of the compartment were positioned closer together and acted as guard rows, with one removed after 8 weeks once the plants had begun to encroach. The middle 6 rows were experimental rows, with 2 per treatment group (Fig. 4c). Slabs held 3 plants and were positioned in gutters that sloped down to a drain at one end of the rows. The plants at the ends of each row were treated as guards and therefore there were 13 experimental plants per row. Plants were supported on wires attached to the roof. For the first 7 days post-transfer of blocks to slabs, the slabs were sealed in plastic wrapping to allow time for the plants to root. Bags were then slashed open at the bottom and dripper lines were attached with 4 per slab, one for each plant and one for the unused position (Supplementary Fig. 3). Dripper lines delivered tomato feed without re-circulation with excess running to waste to avoid cross contamination between treatment groups. Tomato feed composition was confirmed by a water analysis performed by NRM (Bracknell, United Kingdom) (Supplementary Table 1). Feed rate was adjusted based on plant age, but also light levels, with flow increased on days with a higher radiation sum. Environmental conditions were partially controlled - no supplemental heating or lighting was provided; however automatic fans and shades were used to prevent the plants experiencing excess heat and light. The environmental conditions for compartment 2 were: temperature (minimum = 10.5 °C, maximum = 34.1 °C, average = 20.4 °C), relative humidity (minimum = 37.9%, maximum = 100%, average = 77.9%); and for compartment 3: temperature (minimum = 9.8 °C, maximum = 34 °C, average = 20 °C), relative humidity (minimum = 32.8%, maximum = 100%, average = 83.6%). The radiation sum per day was measured via a sensor on the greenhouse roof and was minimum = 346 J cm^−2^, maximum = 2676 J cm^−2^, average = 1379 J cm^−2^.

To limit outbreaks of pests such as whitefly, thrips and aphids, a commercial standard biological control regime was implemented in the trial. Seven days after plants were transferred to stone wool slabs, the following biological control products from Koppert were introduced: En-Strip containing *Encarsia formosa*, Thripor-L containing *Orius laevigatus*, Chrysopa containing *Chrysoperla carnea* and Thripex-V containing *Neoseiulus cucumeris*. Two new Koppert products were added to the biological control regime 120 days after transfer to slabs: Ercal, which contains *Eretmocerus eremicus*, and Spical Ulti-Mite, which contains *Neoseiulus californicus*. The biological control products were replenished as required throughout the trial.

Commercial greenhouse tomato production relies on buzz-pollination by bumble bees for fruit set. To maintain continuity with this practice, pollination was provided by a single buff-tailed bumble bee hive (*Bombus terrestris* ssp. *audax*; Koppert), which was introduced into each greenhouse compartment 3 weeks after plants were transferred to slabs as the first flowers emerged. Supplementary pollen feed was provided in the middle of each compartment to prevent over pollination (Fig. 4c). Hives were replaced every 5/6 weeks to ensure consistent pollination across the trial.

### Vegetative development phenotyping

At 7- and 14-days post sowing, germination was recorded as the percentage of plugs with any part of a seedling visible above the surface. Green leaf area was determined at 18 days post sowing by photographing plants from above and calculating the green leaf area using ImageJ^60^. Height was recorded 49-, 53-, 67- and 71-days post sowing by measuring from the base of the stem to the top of the highest limb.

### Pollination assessment

Bumble bee visitation and pollination of tomato flowers were assessed based on the degree of bruising (brown discoloration) of the anther cone. Five categories of bruising were used as defined previously^62^. Anther cone bruising of all open flowers on the two youngest trusses was recorded for all plants on two sperate occasions 89 and 123 days post sowing, which corresponded to 56 and 90 days post transfer of plants to slabs, respectively. Per plant pollination scores were calculated as $\bar{X(}\left(\sum {Bruising\; level}* {number\; of\; flowers\; on\; truss\; at\; bruising\; level}\right)/{Number\; of\; open\; flowers\; on\; truss})$.

### Tomato fruit phenotyping

Fruit was harvested on a truss-by-truss basis from 98 to 182 days post sowing. In total, twelve trusses were harvested from all plants and fruit mass for each truss was recorded on a per row basis. Additionally, for trusses 6, 8, 9 and 11, at 138-, 153-, 161- and 181-days post sowing, respectively, fruit mass was recorded per plant.

Sugar and acidity of fruit were measured for trusses 6 and 8 at 138- and 153-days post sowing, respectively. For each truss, 3 samples were taken per experimental row, one from each of the three middle slabs. Each sample consisted of 6 fruits, the first pairs of fruit from each of the three plants on one slab. Fruits were blended for 30 s in a juicer (Breville), the juice was passed through miracloth (Millipore, 475855) to remove larger debris and then acidity, sugar (Brix) and sugar/acidity ratio were recorded using Multi Fruits Pocket Brix-Acidity Meter (ATAGO PAL-BX|ACID F5) following the manufacturer’s instructions.

Fruit diameter and ripeness (lycopene content) were determined for all fruit of truss 6 (138 days post sowing). Harvested fruit was photographed on a white background, with a 24 swatch colour checker (Spyder Checkr 24, Datacolor), in a lightbox using a DSLR camera (Nikon D60). Images were processed using a model developed for tomato cv. Tiny Tim that related colour to lycopene content^63^. The model was validated for 20 Arlinta tomatoes by solvent extraction and UV/vis spectrophotometry (Supplementary Fig. 2).

Texture analysis was conducted on fruit from truss 11 (181 days post sowing). Two tomatoes were sampled from 36 plants, one plant from each of the three middle slabs of each experimental row. All texture profiling was conducted according to previous methodology^29^. Briefly, puncture resistance and deformation/resilience tests were performed using a TA-TX plus running Exponent software (v 6.1.27.0) from Stable Micro Systems (Surrey, UK). For both tests pre-test speed was 5 mm s^−1^, test speed was 2 mm s^−1^ and penetration/deformation depth was 5 mm. The system was operated in force trigger mode set at 1 g. The two tests were performed on different fruit. Puncture resistance was measured using a 2 mm diameter steel probe. Three individual tests were performed on each tomato, equidistant from each other and approximately one third of the distance from the bottom end to the equator of the whole fruit. The three parameters given by these tests are: (1) The rupture force (Fr), given by the maximum force during the test; (2) The deformation (Dr) given by the distance moved between the start and the point of rupture; (3) The flesh firmness (FF) given by the average force as the probe is pressed through the flesh.

### Statistical analysis of bioassay and phenotype data

All statistical analysis was performed in R (v 4.4.1). If data showed homoscedasticity and normal distributions, or could be made normal by transformation (Log or Yeo-Johnson), analysis was performed by two-sample *t*-tests (pairwise comparisons) or ANOVAs followed by Tukey post-hoc tests (multiple groups). For pairwise comparisons where the data were heteroscedastic, Welch two-sample *t*-tests were performed. Mann–Whitney tests were performed for pairwise comparisons where the data could not be normalised by transformation. When accounting for repeated measurements, linear mixed-effect models were used. A difference was considered statistically significant at *p* < 0.05.

### Microbial community profiling

Cubes of stone wool, ~1 cm^3^, were collected from three locations: the top of the stone wool block beside the tomato stem, the stone wool slab immediately adjacent to the stone wool block and the middle of the stone wool slab adjacent to the spare dripper line (Supplementary Fig. 3). Algae were scraped from the surface and/or plastic wrapping was cut away prior to collection of the stone wool cubes using sterile scalpels. Stone wool cubes were stored −80 °C.

Stone wool cubes were ground to a powder using liquid nitrogen-cooled pestles and mortars. A total of 20 µl of the ZymoBIOMICS Spike-in Control II (Zymo Research, D6321) was added to ~200 mg of ground material. DNA was extracted using the DNeasy PowerSoil Pro Kit (Qiagen, 47014) and eluted in nuclease-free H_2_O. In addition to the stone wool samples, DNA was also extracted from three replicates of each of the input SynCom inoculums (Laboratory, Commercial and Control) applied to seeds at the start of the trial.

Targeted sequencing of bacterial 16S rRNA genes and fungal ITS2 sequences and surrounding regions of all samples was performed by Zymo Research using their standard protocols. Briefly, sequencing libraries were prepared using the Quick-16S™ NGS Library Prep Kit (Zymo Research, D6400) with primers 515f (GTGYCAGCMGCCGCGGTAA)/806r (GGACTACNVGGGTWTCTAAT)^64,65^ and ITS3 (GCATCGATGAAGAACGCAG)/ITS4 (TCCTCCGCTTATTGATATGC)^66^ for the bacterial 16S rRNA genes and fungal ITS2 sequences, respectively. To avoid amplification of tomato plastid and mitochondrial genes by the 515f/806r primers, PNA PCR blockers (PNA Bio, MP01 and PP01) were included during the library preparation^67^. Libraries were cleaned using Select-a-Size DNA Clean & Concentrator (Zymo Research, D4080) and then quantified by TapeStation (Agilent Technologies) and Qubit (Thermo Fisher Scientific). Sequencing of prepared libraries was carried out on the Illumina NextSeq 1000 platform with P1 Reagent kit.

Sequencing data were provided as FASTQ files. Nextera transposase sequences were removed using Trimmomatic^68^ and reads filtered for quality using a sliding window of 4 bp and a quality score of 20, using ‘keepBothPairs’ to retain both forward and reverse reads. Amplification primers were removed using Cutadapt^69^. Further processing was performed using dada2^70^. Reads were filtered using the default settings (maxEE scores of 2 and 5 for forward and reverse reads, respectively). Error rates were learned using a modified loess function to account for quality score binning in the FastQ files. The weight, span and degree of the loess function was modified and monotonicity enforced as described in the dada2 GitHub issue #1307 (“Binned quality scores and their effect on (non-decreasing) trans rates”)^71^. Forward and reverse reads were merged and chimeras removed. Taxonomic information was assigned using the Silva database (v138.1)^72^ for 16S sequences and the Unite ‘All eukaryotes’ database (v10.0) for ITS sequences^73^. The 16S sequences were aligned using ssu-align^74^ and a phylogenetic tree created using FastTree^75^. Sequences that were categorised as archaeal (max ~1%) or eukaryotic by FastTree were removed from the 16S sequences. Also, although the PNA suppressed amplification of the tomato chloroplast sequences, algal chloroplast sequences were amplified and accounted for up to 50% of the reads. These were also removed. Further analysis was performed using the ‘phyloseq’ package in R^76^. Estimations of bacterial cell counts were calculated using the ZymoBIOMICS Spike-in Control II and following manufacturer’s instructions.

## Supplementary information

Supplementary Information

## Data Availability

Raw sequencing data have been deposited at the European Nucleotide Archive under Accession PRJEB94999. Scripts used for analysis of sequencing data are available from Mendeley Data.
