# Supplementary material for: Synthetic microbial communities for sustainable hydroponic tomato production
Source: NPJ Sustain Agric. 2026 May 26;4(1):42. doi: 10.1038/s44264-026-00147-8 (PMC13212151; doi:10.1038/s44264-026-00147-8)
Supplement: Supplementary file 1 — Supplementary Information [file 44264_2026_147_MOESM1_ESM.pdf]

Synthetic microbial communities for sustainable hydroponic tomato production

Samuel W Wilkinson, Harry C Wright, T E Anne Cotton, David Pascual-Pardo, Stuart A Campbell, Upuli Wickramaarachchi, Duncan D Cameron, Boglarka Z Gulyas, Peter Ho, Alan R Mackie, Samantha J Caton, Jurriaan Ton, Stephen A Rolfe

Supplementary Information

Supplementary Figures

Supplementary Fig. 1 - Testing Synthetic communities (SynComs) of plant-beneficial microbes with commercial tomato variety.

Supplementary Fig. 2 - Training model to quantify lycopene content in the fruit of the tomato cultivar Arlinta from images.

Supplementary Fig. 3 - Commercial demonstration trial stone wool sampling locations.

Supplementary Fig. 4 - Profile of the stone wool microbiome at the end of a 6-month tomato growth trial.

Supplementary Tables

Supplementary Table 1 - Tomato feed analysis.

Supplementary Table 2 - Tomato performance parameters under simulated commercial conditions.

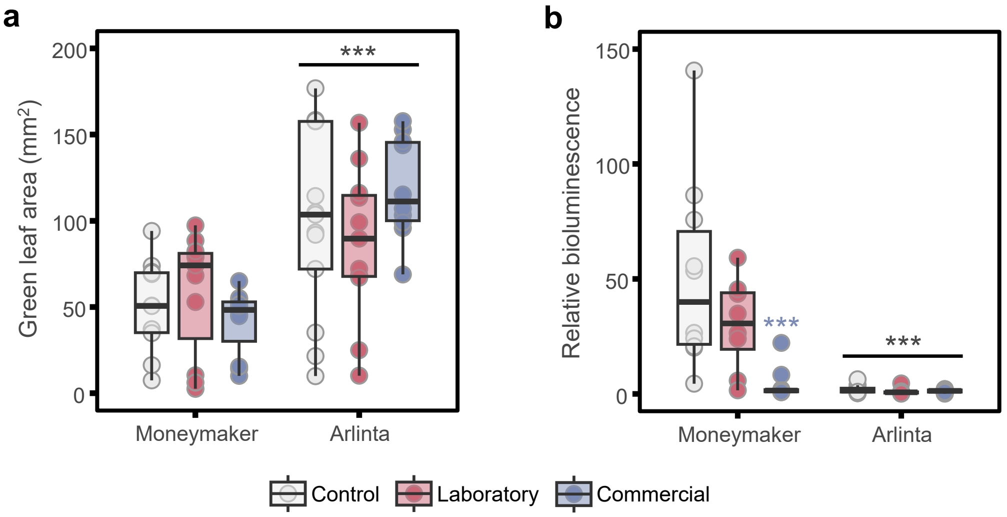

Supplementary Fig. 1 | Testing Synthetic communities (SynComs) of plant-beneficial microbes with commercial tomato variety. a,b, growth and disease resistance seedling trials with MoneyMaker and commercial tomato cv. Arlinta inoculated at sowing with a 3-member SynCom (Laboratory SynCom: *Clonostachys rosea* J1446, *Clonostachys rosea* IK726 and *Pseudomonas chlororaphis* PCL1391; Commercial SynCom: *Clonostachys rosea* J1446, *Trichoderma harzianum* T22 and *Bacillus subtilis* QST713) or water control. *Pst::LUX* colonisation of tomato plants (**b**) was quantified 3 days post inoculation (n = 8-12). Asterisks indicate a significant different between SynComs and control for a particular genotype (green/red) or differences between genotypes (black) as determined by two tailed t-test (*** p < 0.001).

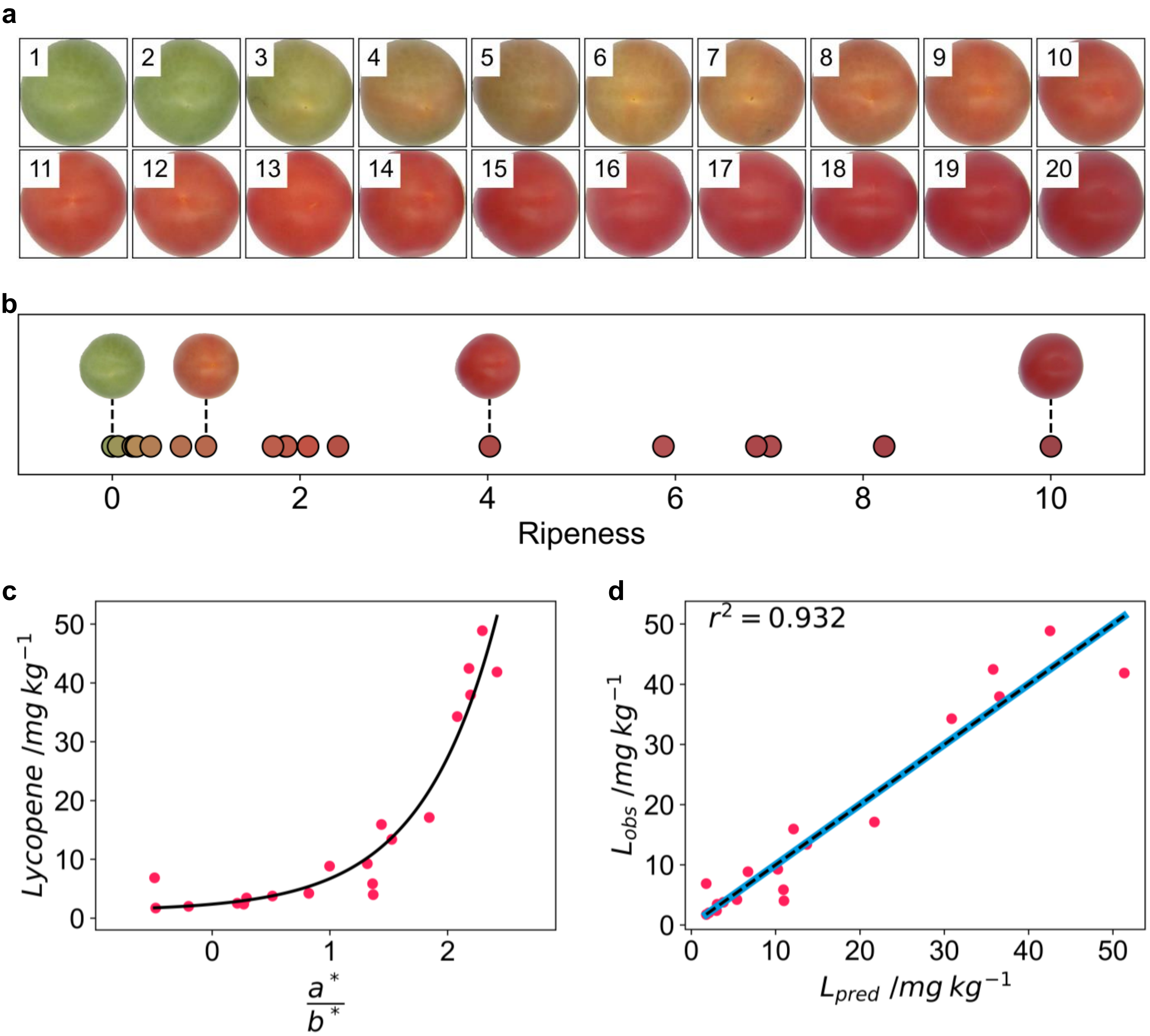

Supplementary Fig. 2 | Training model to quantify lycopene content in the fruit of the tomato cultivar Arlinta from images. a, The 20 fruit used to train the Arlinta version of the lycopene model described in Wright et al., 2023. Images are background and colour corrected and show the fruit prior to extraction of lycopene. **b**, normalised ripeness scale for the 20 fruit. **c**, Lycopene content, as determined by extraction and spectrophotometry, against a* / b* values which are colour channels in the CIELAB colour space. The black line indicates an exponential fit of the data used to predict lycopene content. **d**, Observed lycopene content, as determined by extraction and spectrophotometry, against predicted lycopene content, as determined by the lycopene model using the CIELAB colour space. The black dashed line indicates the y = x line and the r^2 of the fit is inset and the solid blue line shows the fit of the observed and predicted data.

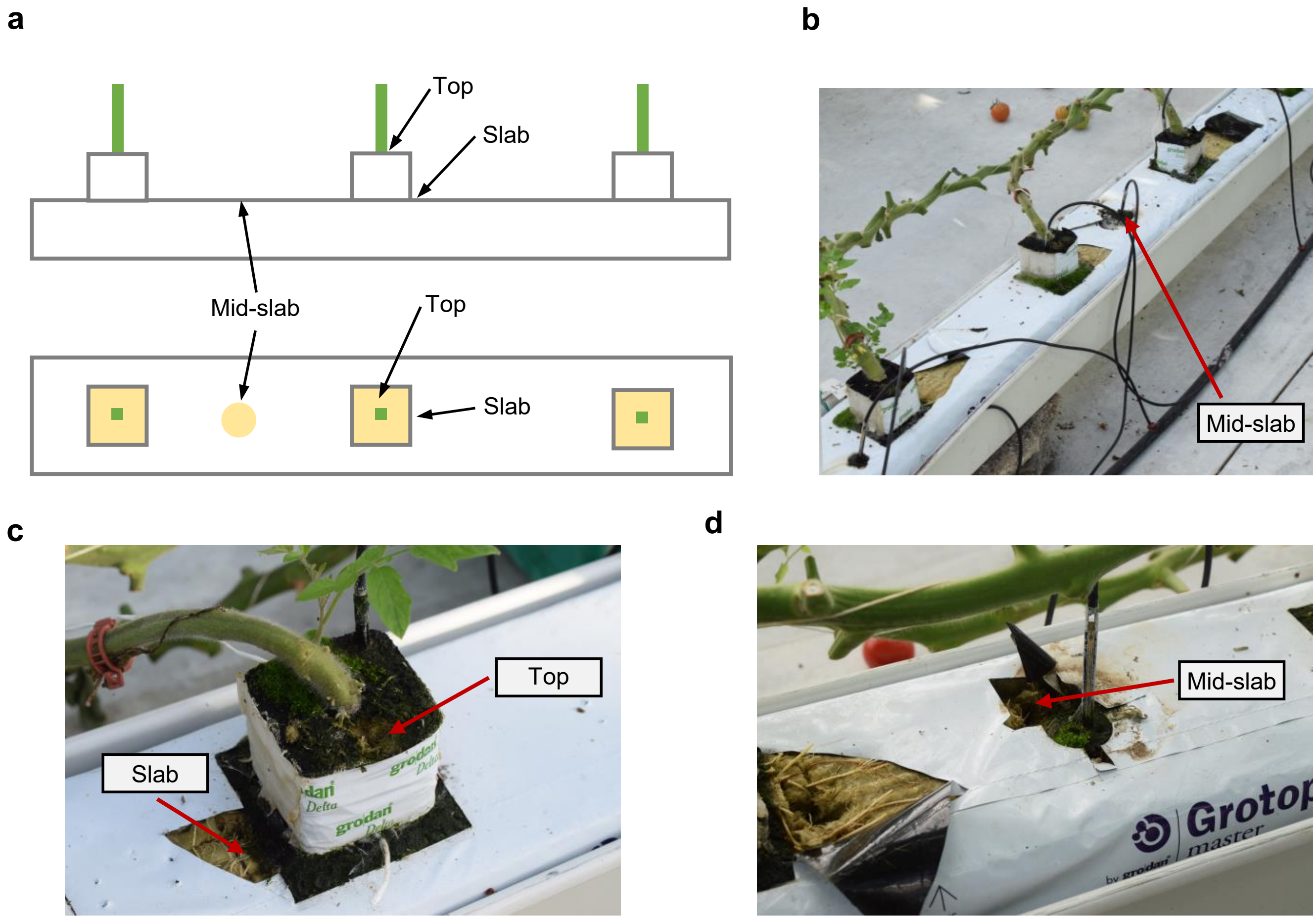

Supplementary Fig. 3 | Commercial demonstration trial stone wool sampling locations. a, Layout of one stone wool slab from the side (upper diagram) and top (lower diagram). Stone wool was sampled for microbiome profiling, from three locations labelled top, slab and mid-slab. **b,c,d,** images of the three rockwool sampling locations.

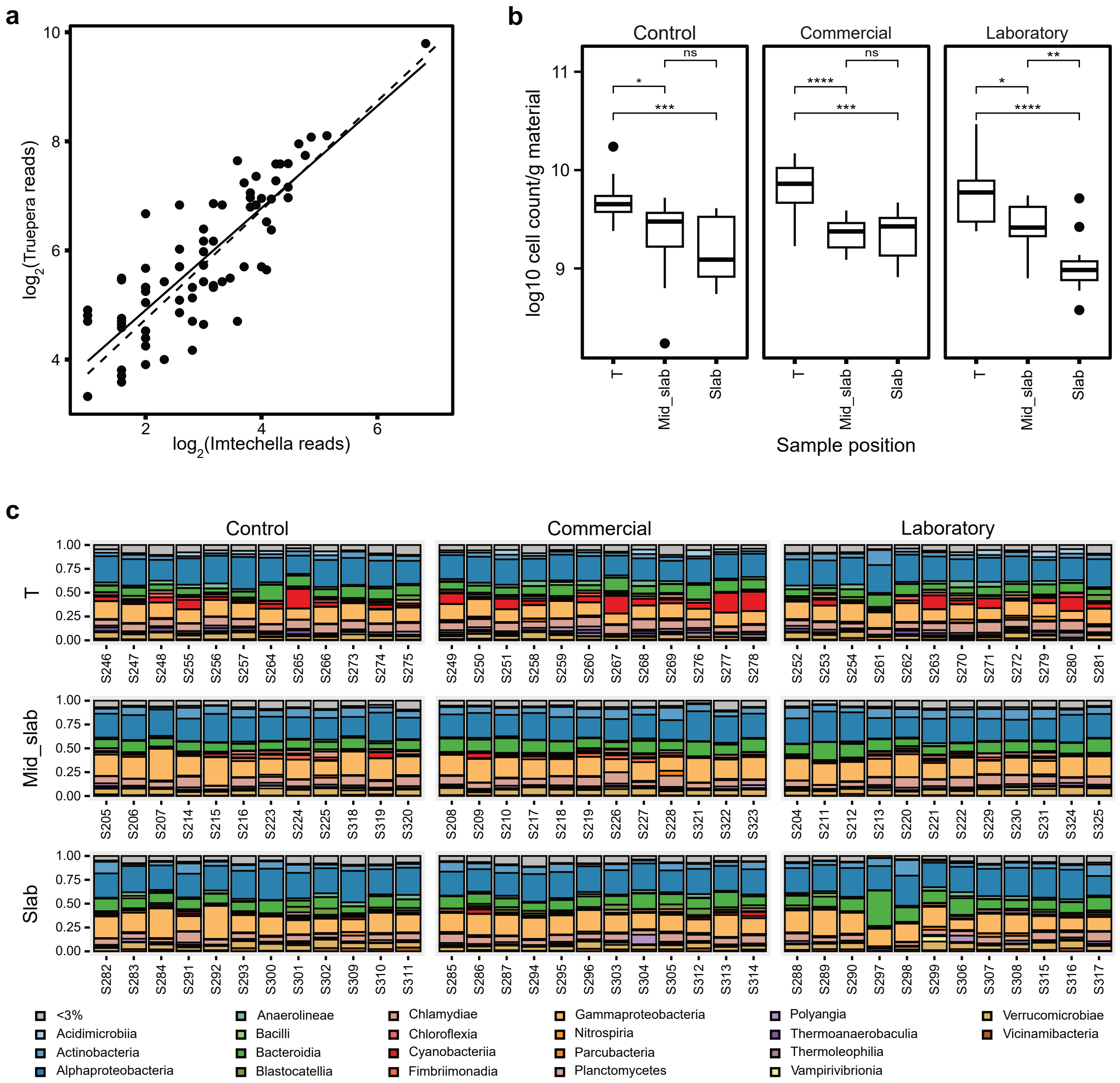

Supplementary Fig. 4 | Profile of the stone wool microbiome at the end of a 6-month tomato growth trial. a, relationship between reads assigned to two of the ZymoBIOMICS Spike-in Control II (Low Microbial Load) bacteria strains, *Truepera radiovictrix* and *Imtechella halotolerans.* Solid black line depicts a 1-1 relationship, and the dashed line depicts the actual linear regression. **b**, bacterial numbers estimated from the 108 16s rRNA amplicon sequencing samples gathered from: next to the tomato stem base (T), in the stone wool slab next to the base of the stone wool cube (Slab) and in the middle of the stone wool slab (Mid_slab). Asterisks indicate a significant difference between sampling positions as determined by two-tailed t-test (* p < 0.05; ** p < 0.01; *** p < 0.001; ns = not significant). Lower, middle and upper horizontal lines in boxplots indicate the 1st, 2nd and 3rd quartiles; whiskers extend to the lowest and highest data points within 1.5 × interquartile range below and above the 1st and 3rd quartiles. **c**, Relative abundances of bacteria at the Class taxonomic level for all 108 16s rRNA amplicon sequencing samples. Classes with a relative abundance less than 3% are grouped together.

Supplementary Table 1 – Tomato feed analysis

Determined	Value	Units
pH	6	
Conductivity	1604	uS/cm
Nitrate-N	154.7	mg/l
Chloride	32.4	mg/l
Sulphate as SO_4	192.1	mg/l
Phosphorus as P	18.3	mg/l
Boron	0.16	mg/l
Potassium	200.1	mg/l
Magnesium	37.34	mg/l
Calcium	155.8	mg/l
Sodium	18.6	mg/l
Carbonate	< 10	mg/l
Alkalinity as HCO_3	< 10	mg/l
TDS	1709.26	mg/l

Supplementary Table 2 – Tomato performance parameters under simulated commercial conditions

**Parameter**	**Measurement timepoint (days post sowing)**	**Unit**	**Control Mean ± SEM***	**Laboratory SynCom Mean ± SEM***	**Commercial SynCom Mean ± SEM***	**Ctrl Vs Laboratory SynCom – Statistical analysis descriptives**	**Ctrl Vs Commercial SynCom – Statistical analysis descriptives**
Germination	7	%	49.3	40.0	33.3	N/A	N/A
Germination	14	%	90.7	88.0	84.0	N/A	N/A
Green leaf area	18	cm^2	8.95 ± 0.38	5.41 ± 0.24***	4.57 ± 0.27***	Welch's t-test: t = 8.18, df = 216.15, $p.adj$ < 0.001	Welch's t-test: t = 10.69, df = 188.60, $p.adj$ < 0.001
Height	49	cm	63.27 ± 0.46	59.44 ± 0.83**	53.38 ± 1.08***	Mann–Whitney: U = 1812, n_1 = 52, n_2 = 52, $p.adj$ < 0.01	Mann–Whitney: U = 2323, n_1 = 52, n_2 = 52, $p.adj$ < 0.001
Height	53	cm	71.38 ± 0.47	66.33 ± 0.60***	63.08 ± 0.83***	Welch's t-test: t = 6.65, df = 96.26, $p.adj$ < 0.001	Welch's t-test: t = 8.68, df = 80.16, $p.adj$ < 0.001
Height	67	cm	101.33 ± 0.64	96.33 ± 0.97***	91.13 ± 0.93***	Mann–Whitney: U = 2006.5, n_1 = 52, n_2 = 52, $p.adj$ < 0.001	Mann–Whitney: U = 2358.8, n_1 = 52, n_2 = 52, $p.adj$ < 0.001
Height	71	cm	117.37 ± 0.75	113.44 ± 0.97***	107.83 ± 0.96***	Mann–Whitney: U = 1905.0, n_1 = 52, n_2 = 52, $p.adj$ < 0.001	Mann–Whitney: U = 2298.5, n_1 = 52, n_2 = 52, $p.adj$ < 0.001

Pollination	89	Pollination score (0-4)	1.94 ± 0.08	2.10 ± 0.10	1.98 ± 0.10	Welch's t-test: *t* = -1.32, df = 98.17, *p.adj* = 0.38	Welch's t-test: *t* = -0.35, df = 95.30, *p.adj* = 0.72
Pollination	123	Pollination score (0-4)	2.44 ± 0.14	2.43 ± 0.16	2.27 ± 0.14	Mann–Whitney: *U* = 1281.5, n_1 = 50, n_2 = 50, *p.adj* = 0.83	Mann–Whitney: *U* = 1261.5, n_1 = 50, n_2 = 46, *p.adj* = 0.83
Total fruit yield	98-182	kg	343.87	344.56	335.96	N/A	N/A
Row fruit yield	98-182	kg	7.16 ± 0.20	7.18 ± 0.14	7.00 ± 0.17	Full model: Mass ~ Treatment + (1|Truss)<br>Reduced model: Mass ~ 1 + (1|Truss)<br>X^2 = 1.516, df = 2, *p* = 0.4686	
Ripeness – truss 6	138	Normalised ripeness (0-10)	5.14 ± 0.26	5.00 ± 0.25	4.06 ± 0.21**	T-test: *t* = 0.39, df = 98, *p.adj* = 0.70	T-test: *t* = 3.27, df = 98, *p.adj* < 0.01
Fruit diameter – truss 6	138	cm	4.60 ± 0.04	4.58 ± 0.03	4.62 ± 0.04	T-test: *t* = 0.35, df = 98, *p.adj* = 0.76	T-test: *t* = -0.31, df = 98, *p.adj* = 0.76
Plant Fruit Mass – truss 6	138	g	547.92 ± 17.69	572.00 ± 13.50	576.66 ± 16.44	Mann–Whitney: *U* = 1188.5, n_1 = 50, n_2 = 51, *p.adj* = 0.56	Mann–Whitney: *U* = 1120.0, n_1 = 50, n_2 = 50, *p.adj* = 0.56
Fruit Brix – truss 6	138	%	6.12 ± 0.11	5.82 ± 0.13	5.93 ± 0.13	T-test: *t* = 1.76, df = 22, *p.adj* = 0.18	T-test: *t* = 1.14, df = 22, *p.adj* = 0.27

Fruit Acidity – truss 6	138	%	0.55 ± 0.01	0.54 ± 0.01	0.53 ± 0.02	T-test: t = 0.70, df = 22, $p.adj$ = 0.49	T-test: t = 1.07, df = 22, $p.adj$ = 0.49
Fruit brix/acidity – truss 6	138	N/A	11.10 ± 0.21	10.80 ± 0.18	11.19 ± 0.29	T-test: t = 1.11, df = 22, $p.adj$ = 0.56	T-test: t = -0.25, df = 22, $p.adj$ = 0.81
Plant Fruit Mass – truss 8	153	g	582.92 ± 14.40	561.61 ± 13.29	590.90 ± 19.19	Mann–Whitney: U = 1333.5, n_1 = 51, n_2 = 49, $p.adj$ = 0.57	Mann–Whitney: U = 1079.5, n_1 = 51, n_2 = 50, $p.adj$ = 0.37
Fruit Brix – truss 8	153	%	5.78 ± 0.06	5.85 ± 0.06	5.82 ± 0.09	Mann–Whitney: U = 57.5, n_1 = 12, n_2 = 12, $p.adj$ = 0.79	Mann–Whitney: U = 67, n_1 = 12, n_2 = 12, $p.adj$ = 0.79
Fruit Acidity – truss 8	153	%	0.47 ± 0.01	0.50 ± 0.01	0.49 ± 0.01	T-test: t = -1.68, df = 22, $p.adj$ = 0.22	T-test: t = -0.83, df = 22, $p.adj$ = 0.42
Fruit brix/acidity – truss 8	153	N/A	12.28 ± 0.23	11.84 ± 0.29	12.02 ± 0.23	T-test: t = 1.15, df = 22, $p.adj$ = 0.45	T-test: t = 0.78, df = 22, $p.adj$ = 0.45
Plant Fruit Mass – truss 9	161	g	576.10 ± 16.16	583.88 ± 17.36	595.19 ± 15.83	Mann–Whitney: U = 1280.5, n_1 = 50, n_2 = 50, $p.adj$ = 0.84	Mann–Whitney: U = 1137.0, n_1 = 50, n_2 = 48, $p.adj$ = 0.84
Plant Fruit Mass – truss 11	181	g	656.33 ± 20.75	634.74 ± 11.64	668.27 ± 15.37	T-test: t = 0.91, df = 97, $p.adj$ = 0.65	T-test: t = -0.46, df = 96, $p.adj$ = 0.65

Post-harvest disease resistance: lesion diameter 3 dpi – truss 11	181	mm	8.69 ± 0.73	7.81 ± 0.79	7.80 ± 0.64	T-test: t = 0.82, df = 62, $p.adj$ = 0.42	T-test: t = 0.91, df = 62, $p.adj$ = 0.42
Post-harvest disease resistance: lesion expansion rate 3 to 5 dpi – truss 11	181	mm/day	4.67 ± 0.67	3.04 ± 0.67	3.15 ± 0.64	T-test: t = 2.02, df = 54, $p.adj$ = 0.10	T-test: t = 1.67, df = 55, $p.adj$ = 0.10
Fruit rupture force – truss 11	181	N	39.6 ± 1.2	41.5 ± 2.0	39.2 ± 1.5	One-way Anova: F = 1.54, df = 2, p = 0.22	
Fruit deformation to rupture – truss 11	181	mm	3.1 ± 0.1	3.2 ± 0.2	2.9 ± 0.1	One-way Anova: F = 1.26, df = 2, p = 0.29	
Fruit flesh firmness – truss 11	181	N	5.5 ± 0.3	5.6 ± 0.3	5.9 ± 0.3	One-way Anova: F = 0.45, df = 2, p = 0.64	

* Data displayed are means ± SEM unless treatment totals are provided.
